# Associations of age at type 2 diabetes diagnosis with ethnicity and socioeconomic deprivation: observational study in 11.5 million people in England

**DOI:** 10.1186/s12939-026-02924-w

**Published:** 2026-06-10

**Authors:** Jonathan Goldney, Sharmin Shabnam, Mary M. Barker, Jack A. Sargeant, Enya Daynes, Louise M. Goff, Kamlesh Khunti, Joseph Henson, Melanie J. Davies, Francesco Zaccardi

**Affiliations:** 1 Diabetes Research Centre, College of Life Sciences, University of Leicester, Leicester General Hospital, Leicester, LE5 4PW UK; 2 NIHR Leicester Biomedical Research Centre, University Hospitals of Leicester NHS Trust and University of Leicester, Leicester, LE5 4PW UK; 3 Leicester Real World Evidence Unit, Leicester Diabetes Centre, University of Leicester, Leicester General Hospital, Leicester, LE5 4PW UK; 4 Unit of Integrative Epidemiology, Institute of Environmental Medicine, Karolinska Institutet, Stockholm, Sweden; 5 Leicester Diabetes Centre, University Hospitals of Leicester NHS Trust, Leicester, LE5 4PW UK; 6 Department of Respiratory Sciences, University of Leicester, Glenfield Hospital, Leicester, LE3 9QP UK; 7 NIHR Applied Research Collaboration East Midlands (ARC-EM), Leicester Diabetes Centre, University of Leicester, Leicester, LE5 4PW UK

**Keywords:** Early-onset, Type 2 diabetes, Ethnicity, Deprivation, Intersectionality, Inequality

## Abstract

**Background:**

In England, younger individuals with type 2 diabetes (T2D) are more likely to be from minority ethnic groups and from higher levels of socioeconomic deprivation as compared to older individuals with T2D. It remains unclear whether younger individuals from these groups are at a heightened risk of T2D, or whether the observed distribution of ethnicity, deprivation, and age among those with T2D reflects that of the general population.

**Methods:**

In this cross-sectional study, we used the Clinical Practice Research Datalink to identify adults with newly-diagnosed T2D, matched to up to 5 individuals without, in England between Jan 2000-Mar 2021. We estimated the distribution of individuals with T2D across ethnic groups and deprivation quintiles by age at diagnosis, as compared to individuals without T2D. Proportion ratios were calculated by dividing the proportion with T2D from each ethnic/deprivation group by the proportion without T2D from the corresponding group.

**Results:**

We included 2,676,039 individuals with T2D and 8,987,347 without. Across age, the pattern in the proportions of individuals with T2D from each ethnic group closely resembled the patterns in individuals without T2D, although there were consistently higher proportions from Black, South Asian, and other ethnic groups, meaning that the proportion ratio of being from these groups (comparing with vs without T2D) was consistently > 1. Nevertheless, proportion ratios for Black or other ethnic groups generally increased with age, being more stable for South Asian ethnicity. The proportion ratios for White ethnicity were consistently < 1; however, the pattern was U-shaped across age.

At most ages, White individuals with T2D were more likely to be from the most deprived quintile and less likely to be from the least deprived quintile compared to individuals without T2D. There was generally no associations between T2D status and deprivation in individuals from minority ethnic groups across age.

**Conclusions:**

Our findings suggest that minority ethnicity status is a consistent risk factor for T2D across all age groups, rather than being disproportionately elevated at younger ages. The varying associations between T2D and deprivation across ethnic groups requires further investigation, particularly to explore whether T2D may be underdiagnosed in individuals from minority ethnic groups who experience high levels of deprivation.

**Supplementary information:**

The online version contains supplementary material available at 10.1186/s12939-026-02924-w.

## Background

In Western countries, the incidence of type 2 diabetes (T2D) is now stable or even falling [1]; however, in both the UK and globally, the incidence of early-onset T2D (EOT2D; diagnosis age < 40 years) continues to rise [2, 3]. EOT2D is associated with an increased burden of complications compared to a later-onset, in particular microvascular disease, as well as increased risk of mortality and a shorter life expectancy [4–7]. EOT2D is also associated with a significant psychological burden [8, 9], alongside stigma and a range of barriers to healthcare and well-being [10]. Understanding risk factors for EOT2D is therefore important in developing preventative strategies to reduce the incidence of T2D in early adulthood.

It is well established that, in Western countries, individuals from minority ethnic groups have a higher risk of T2D as compared to White individuals [11–13]. Furthermore, the risk of T2D associated with ethnicity may be even greater with a younger age at diagnosis, suggested by particularly large proportions of individuals with EOT2D from minority ethnic groups across several countries, including Māori or Pasifika ethnicity in New Zealand (71% of individuals < 40 years of age from these groups) and Pima Indian ethnicity in the USA (~5% of individuals from Pima Indian ethnicity aged 35–45 years have prevalent T2D) [14, 15]. Furthermore, in England, data from the National Diabetes Audit (NDA) highlights that younger individuals with T2D are more likely to be from South Asian, Black, Mixed or other ethnic groups as compared to older individuals with T2D [16]: for example, the proportion from these groups was 51% <16 years of age, 41% at 16–18 years, 38% at 19–25 and 26–39 years, 27% at 40–59 years and 15% at 60–79 years. It is unknown, however, to what extent these varying proportions may simply reflect the age-specific distribution of ethnicity in individuals without T2D, or whether younger individuals from minority ethnic backgrounds are at an increased risk of T2D as compared to older individuals.

Similarly, socioeconomic deprivation is a well-established risk factor for T2D in Western countries [17]. As with ethnicity, large population-wide studies are lacking to determine whether deprivation is associated with an increased risk of EOT2D in England, as compared to later-onset T2D. Again, data from the English NDA demonstrates that younger individuals with T2D are more likely to be from the most deprived quintile of deprivation, and less likely to be from the least deprived quintile, as compared to older adults [16]. For example, the proportion of individuals from the most deprived quintile was 42% among those under 16 years, decreasing progressively with age: 38% at 16–18 years, 35% at 19–25 and 26–39 years, 29% at 40–59 years and 22% at 60–79 years. It remains unclear whether these trends reflect the age-specific distribution of deprivation in the general population, or whether deprivation is a stronger risk factor for T2D at younger ages. Given that the association between deprivation and T2D status varies in both strength and direction across ethnic groups [18, 19], further research is needed to explore potential interactions in risk.

Indeed, there is increasing recognition that intersectional approaches to health research are required. Intersectionality is a theoretical framework which recognises that multiple overlapping social characteristics shape an individual’s position within society. Health systems may overlook these overlapping characteristics, which alongside the interplay of systematically discriminated identities, may influence access to healthcare and health outcomes. Whilst quantitative approaches cannot fully capture all intersecting characteristics which define an individual’s social position, the importance of understanding intersections in health outcomes across multiple disadvantaged or discriminated characteristics (e.g., age, sex, ethnicity, deprivation) is increasingly recognised.

Given these gaps in the literature, we set out to investigate in a large, representative nationwide cohort how ethnicity and deprivation vary with age at diagnosis in individuals with T2D as compared to individuals without T2D. We applied an intersectional approach by stratifying across multiple intersecting social positions- age, sex, T2D status, ethnicity and deprivation- allowing exploration of differences across social intersections without imposing model-based assumptions about the form of interactions.

We hypothesised that the proportion of individuals with T2D from minority ethnic groups and/or higher levels of deprivation disproportionately increases relative to the general population with a younger age at diagnosis. This would suggest that the increased risk of T2D associated with minority ethnic status and/or higher levels of deprivation may be higher at younger ages.

## Methods

To investigate why high proportions of individuals with EOT2D are from minority ethnic/high deprivation groups relative to later-onset T2D, we undertook a cross-sectional study, to allow comparison of ethnicity/deprivation population composition across T2D status and age.

### Data source

This cross-sectional study used data from the Clinical Practice Research Datalink (CPRD) GOLD and Aurum, which contain anonymised electronic healthcare record (EHR) data from a large representative sample of UK-wide primary care practices. The data represents over 2,000 primary care practices and 60 million individuals, 18 million of which are currently registered active patients, and includes information on demographics and diagnoses [20]. This study was approved by the CPRD Independent Scientific Advisory Committee (protocol 23_002869) and has been reported in line with the RECORD guidelines (checklist in the Supplementary Material).

### Study population

All individuals aged ≥ 18 years with their first-ever diagnostic code for T2D recorded in CPRD between 1 January 2000 and March 2021 were identified. A cohort without T2D was also identified by matching individuals with T2D to up to 5 individuals without T2D according to year of birth, sex, and primary care practice. Individuals without T2D were identified on the same date as individuals with newly-diagnosed T2D that they were matched to. The matched cohort also did not include individuals with any previous diabetes code related to type 1 diabetes. We also excluded duplicate records for both CPRD GOLD and Aurum; missing data for sex, ethnicity, or deprivation; and records without available linkage to Hospital Episode Statistics (HES; with information on diagnoses, procedures and demographic data for hospitalised patients) and Office for National Statistics (ONS; with information on date and cause of death) data; HES and ONS were available only for England. The cohort definition is shown in Supplementary Figure S1.

### Demographic factors

Ethnicity was defined using the following categories and subcategories in HES data: Black (including Black African, Black Caribbean, Black Other), South Asian (Bangladeshi, Indian, Pakistani), White, and Other (Chinese, Other Asian, Mixed, Other). As ethnicity is self-reported and socially-defined, it was therefore not treated as a biological characteristic. Whilst associations between ethnicity and health outcomes may be influenced by genetic factors, they also reflect social processes including discrimination and structural racism. We did not make a priori assumptions regarding the relative contribution of these mechanisms, and ethnicity was included in line with its use in previous literature.

Deprivation status was identified using linked Index of Multiple Deprivation (IMD) 2019 data, which was categorised into five quintiles (1^st^ quintile, least deprived; 5^th^ quintile, most deprived) [21]. IMD measures multiple deprivation at a small area level, compromising between 400 and 1200 households and approximately 1000–3000 individuals [22], and reflects seven different domains: income deprivation, employment deprivation, health deprivation and disability, education skills and training deprivation, barriers to housing and services, living environment deprivation, and crime. Sex was extracted from CPRD.

### Statistical analysis

Given the very large sample, we grouped age into 14 categories: 18–24 years, categories spanning 5-years between ages 25 and 84 (i.e., 25–29, 30–34, …), and ≥85 years. Across T2D status and sex, we summarised age at T2D diagnosis (or matched age in those without diabetes), ethnicity and deprivation: variables were presented as number (%) or median (interquartile range [IQR]), as appropriate. Given that approximately 25% of the cohort had missing data for ethnicity and were excluded (Figure S1), we summarised characteristics across this cohort to explore differences which could influence external and internal validity.

An intersectional analytic approach was adopted through stratification rather than interaction or other modelling strategies. Separately in individuals with and without T2D, we calculated the proportion of individuals from each of the four ethnic groups (across age categories and deprivation) and from each quintile of deprivation (across age categories and ethnicity): the Wilson method was used to obtain the confidence intervals (CIs). We also estimated the proportion ratios (with vs without T2D) for ethnicity and deprivation, by dividing the proportion of individuals from each ethnic/deprivation group in those with T2D by the corresponding proportion in individuals without T2D [23]. Proportion ratios provide a single figure that describes whether the proportion of individuals from each ethnicity/deprivation group is higher in individuals with T2D (proportion ratio > 1) or without T2D (proportion ratio < 1).

All analyses were stratified by sex and undertaken in StataBE (v18.0) using the University of Leicester High Performance Computing (ALICE) and results reported with 95% confidence intervals (CIs). Aggregate results for all analyses and the codes used for the initial data cleaning process and the study analysis are available at github (jgoldney).

## Results

### Cohort characteristics

The study cohort included 2,676,039 individuals with newly-diagnosed T2D (53.8% women; median age: 55 [IQR: 41–70] years in women and 58 [46–69] years in men]) matched to 8,987,347 individuals without T2D (54.9% women; median age: 53 [38–69] and 56 [45–68] years in women and men, respectively). The characteristics of the study cohort are reported in Table 1, stratified by sex and T2D status.

**Table 1:** Characteristics of the cohort

		Women		Men	
		Individuals with type 2 diabetes	Individuals without type 2 diabetes	Individuals with type 2 diabetes	Individuals without type 2 diabetes
No. of people		1,439,510 (12.3)	4,932,240 (42.3)	1,236,529 (10.6)	4,055,107 (34.8)
Age (years)		55 (41, 70)	53 (38, 69)	58 (46, 69)	56 (45, 68)
Age categories (years)	18–25	66,331 (4.6)	258,130 (5.2)	29,799 (2.4)	112,023 (2.8)
	25–29	74,826 (5.2)	302,462 (6.1)	33,071 (2.7)	126,789 (3.1)
	30–34	91,574 (6.4)	374,463 (7.6)	46,417 (3.8)	176,758 (4.4)
	35–39	97,227 (6.8)	389,518 (7.9)	62,614 (5.1)	234,651 (5.8)
	40–44	117,013 (8.1)	440,553 (8.9)	95,385 (7.7)	348,459 (8.6)
	45–49	122,243 (8.5)	427,017 (8.7)	116,943 (9.5)	411,107 (10.1)
	50–54	127,936 (8.9)	416,084 (8.4)	136,305 (11.0)	454,768 (11.2)
	55–59	122,717 (8.5)	390,865 (7.9)	140,852 (11.4)	454,733 (11.2)
	60–64	119,477 (8.3)	376,715 (7.6)	140,034 (11.3)	439,941 (10.8)
	65–69	120,680 (8.4)	373,884 (7.6)	134,305 (10.9)	411,970 (10.2)
	70–74	115,791 (8.0)	360,352 (7.3)	114,277 (9.2)	347,935 (8.6)
	75–79	100,052 (7.0)	319,862 (6.5)	85,965 (7.0)	262,540 (6.5)
	80–84	80,727 (5.6)	262,022 (5.3)	57,709 (4.7)	171,631 (4.2)
	≥85	82,916 (5.8)	240,313 (4.9)	42,853 (3.5)	101,802 (2.5)
Ethnicity	Black	99,807 (6.9)	269,577 (5.5)	65,477 (5.3)	163,898 (4.0)
	South Asian	97,645 (6.8)	215,847 (4.4)	74,672 (6.0)	147,766 (3.6)
	White	1,153,278 (80.1)	4,174,589 (84.6)	1,024,329 (82.8)	3,551,528 (87.6)
	Other	88,780 (6.2)	272,227 (5.5)	72,051 (5.8)	191,915 (4.7)
Quintile of deprivation	1^st^- Least deprived	251,094 (17.4)	886,948 (18.0)	220,677 (17.8)	745,091 (18.4)
	2^nd^	265,876 (18.5)	942,467 (19.1)	236,434 (19.1)	795,240 (19.6)
	3^rd^	283,585 (19.7)	979,808 (19.9)	245,082 (19.8)	806,426 (19.9)
	4^th^	328,012 (22.8)	1,116,760 (22.6)	272,070 (22.0)	875,532 (21.6)
	5^th^- Most deprived	310,943 (21.6)	1,006,257 (20.4)	262,266 (21.2)	832,818 (20.5)
Year of identification		2013 (2008, 2017)	2013 (2007, 2017)	2012 (2007, 2016)	2011 (2006, 2016)

Data are presented as number (%) for categorical variables column-wise, excluding no. of people which is presented row-wise, and as median (interquartile range) for continuous variables

Age indicates the age at diagnosis in individuals with type 2 diabetes and the matched age in those without type 2 diabetes

In women with T2D, 6.9% were from a Black ethnic group as compared to 5.5% without T2D; being 6.8% vs 4.4% for South Asian ethnicity; 80.1% vs 84.6% for White ethnicity; and 6.2% vs 5.5% for other ethnic groups. In men, the pattern was similar: 5.3% vs 4.0% for Black ethnicity with vs without T2D respectively; for South Asian ethnicity, 6.0% vs 3.6%; for White ethnicity, 82.8% vs 87.6%; and for other ethnic groups, 5.8% vs 4.7%. Further breakdown of ethnic subgroups by T2D status and sex is reported in Supplementary Table S1.

In both women and men with and without T2D, more individuals were from the most than the least deprived quintile (Table 1), being 21.6% vs 17.4% respectively in women with T2D, 20.4% vs 18.0% in women without T2D, 21.2% vs 17.8% in men with T2D, and 20.5% vs 18.4% in men without T2D.

The characteristics of individuals with missing ethnicity data are shown in Supplementary Table S2. In general, the cohort with missing data was similar to the primary cohort, although there were proportionally fewer women (43.4% vs 54.6%), more men (56.6% vs. 45.4%), fewer individuals with T2D (15.1% vs 23.9%), more individuals without T2D (84.9% vs 76.1%), and a younger median age (46 vs 55 years), but a similar distribution of deprivation.

### Ethnicity and deprivation in individuals with vs without T2D

#### Ethnicity

**Fig. 1:**
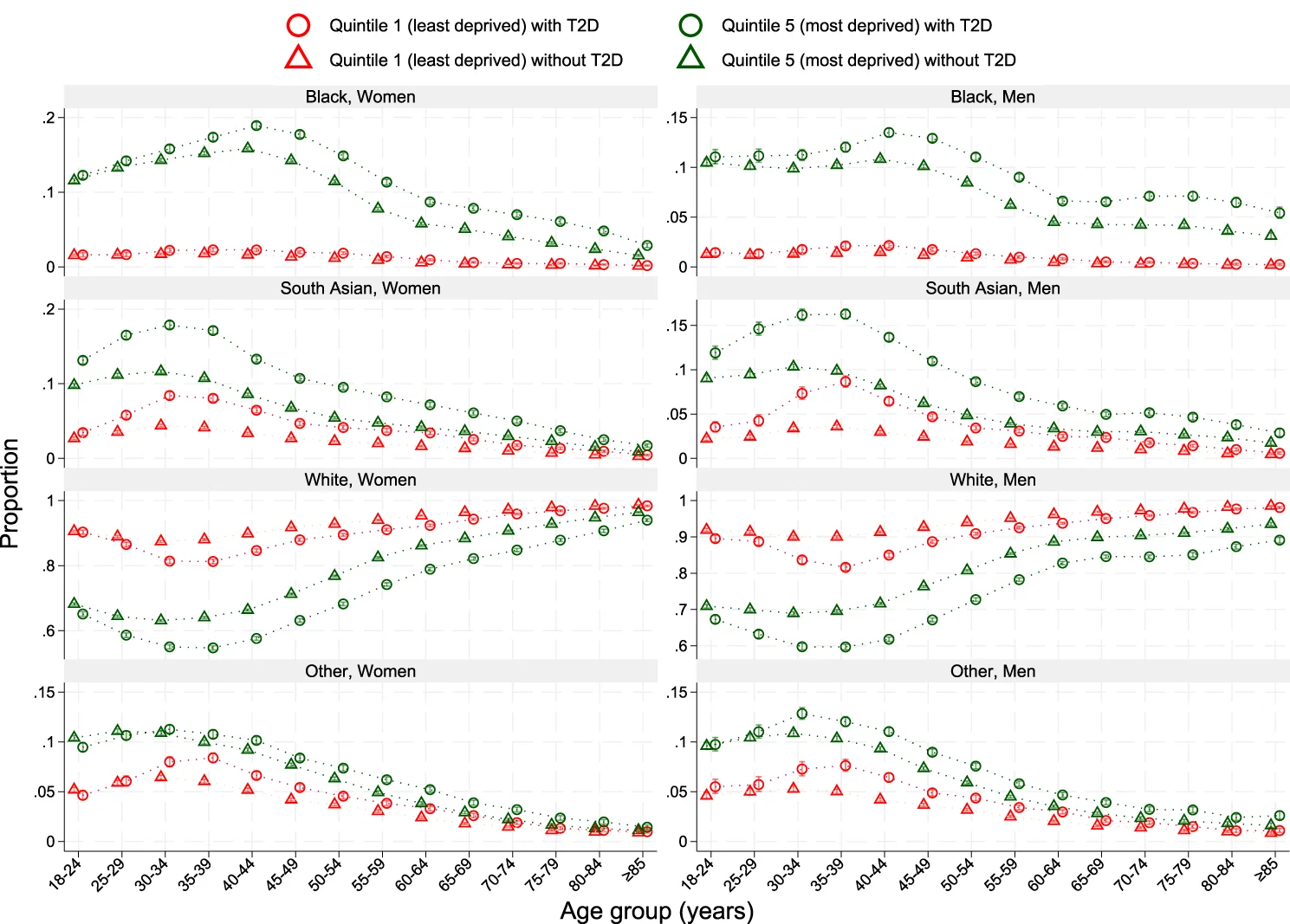
The proportion of women and men from each ethnic group, by diabetes status, age and the most and least deprived quintiles of deprivation. Each plot shows the proportion of women (left panels) and men (right panels) in each ethnicity group across type 2 diabetes (T2D) status (individuals with vs without T2D: circles vs triangles, respectively), quintiles of deprivation (least and most deprived: red and green colours, respectively) and age at T2D diagnosis (x-axis). Note the Y axis range varies by outcome. Estimates are shown with 95% confidence intervals (bars). Dotted lines between estimates were added for visual aid and age was used as a categorical variable, not a continuous

The proportions of individuals from each ethnic group across T2D status, age and the least and most deprived quintiles of deprivation are shown in Figure 1 (proportions across the second, third, and fourth quintiles are shown in Figure S2); the corresponding proportion ratios of being from each ethnic group, comparing individuals with vs without T2D, are shown in Figure S3.

Regardless of deprivation status, the pattern in the proportion from Black, South Asian or other ethnic groups across age in individuals with T2D closely reflected the pattern in individuals without T2D, being highest between ages 30 to 45 years and generally being progressively lower outside this age range (Figs. 1 & S2). In both women and men, across almost all age groups, and regardless of deprivation status, the proportion of individuals from Black, South Asian or other ethnic groups was consistently higher in those with vs without T2D, meaning that the proportion ratio (proportion with T2D divided by the proportion without T2D) was consistently greater than 1 (Figure S3). Despite this, the trend in the proportion ratio with age varied across ethnicities: the proportion ratio for being from Black and other ethnic groups tended to increase with age, particularly for individuals from the most deprived quintiles of deprivation; whereas proportion ratios for South Asian ethnicity were more constant. For example, for Black women in the most deprived quintile of deprivation, the proportion ratio increased with age from a minimum of 1.06 (95% CI: 1.01–1.11) at age 18–24 years – reflecting proportions of 0.12 (0.12–0.13) vs 0.12 (0.11–0.12) in women with vs without T2D – to a maximum of 2.02 (1.84–2.22) at age 80–84 years – proportions of 0.05 (0.04–0.05) vs 0.02 (0.02–0.03) with vs without T2D; conversely, proportion ratios for being from a South Asian ethnicity were generally stable between 1.5 and 2.0.

Similarly, regardless of deprivation status, the proportion of individuals with T2D from a White ethnic background closely mirrored that of individuals without T2D, being lowest between ages 30 to 45 years and higher both above and below this range (Figs. 1 & S2). The proportion of individuals from a White ethnic group was consistently lower in those with vs without T2D regardless of age, meaning that proportion ratios for White ethnicity (proportion with T2D divided by the proportion without T2D) were < 1 (Figure S3); however, the difference in the proportions varied across age. Proportion ratios followed a U-shape pattern with increasing age, initially declining with a nadir at the age of 35–39 years and progressively increasing for older ages, regardless of sex or deprivation. At this age, the proportion ratios were 0.92 (95% CI: 0.92–0.93) and 0.85 (0.84–0.87) in women from the least and most deprived quintiles respectively, while corresponding figures in men were 0.91 (0.9–0.92) and 0.86 (0.85–0.87). For both sexes at extremes of age proportion ratios were closer to 1.

#### Deprivation

**Fig. 2:**
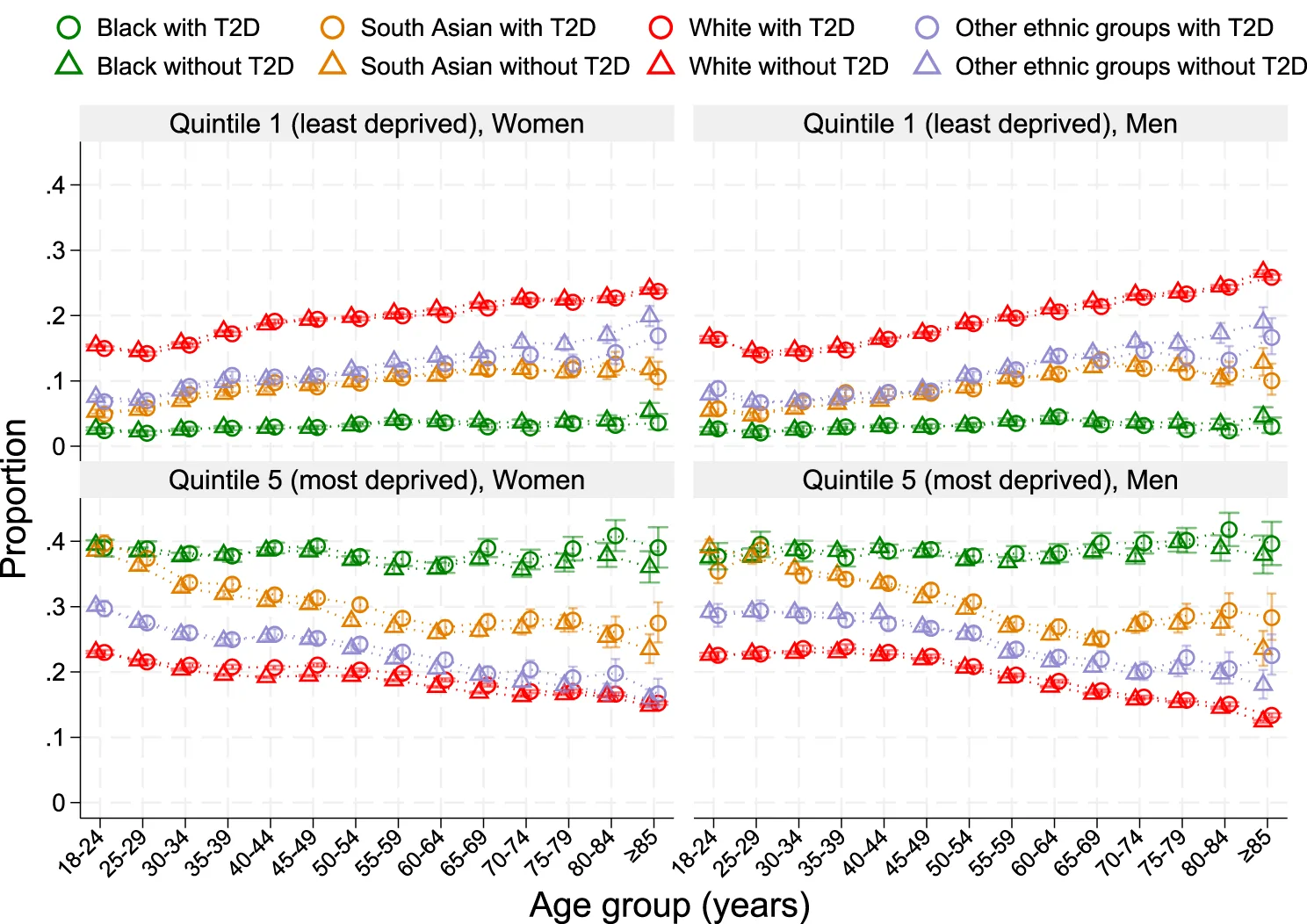
The proportion of women and men from the least and most deprived quintile of deprivation, by diabetes status, age and ethnicity. Each plot shows the proportion of women (left panels) or men (right panels) from the least (quintile 1) and most (quintile 5) deprivation group across type 2 diabetes (T2D) status (individuals with vs without T2D: circles vs triangles, respectively), ethnicity (Black, green; South Asian, orange; White, red; Other, lavender) and age at T2D diagnosis (x-axis). Note the Y axis range varies by outcome. Estimates are shown with 95% confidence intervals (bars). Dotted lines between estimates were added for visual aid and age was used as a categorical variable, not a continuous

The proportion of individuals from the least and most deprived quintiles by T2D status, age and ethnicity are shown in Fig. 2 (with the remaining quintiles shown in Figure S4); the corresponding proportion ratios of being from the least and most deprived quintiles, comparing individuals with vs without T2D, are shown in Figure S5 (proportion ratios for the remaining quintiles shown in Figure S6).

Across sex, age, and ethnicity, the patterns in the proportion of individuals with T2D from each deprivation quintile closely resembled the patterns in individuals without T2D (Fig. 2 & S4). In both women and men from South Asian, White, and other ethnic groups, there was a progressive increase in the proportion of those from the least deprived quintile with increasing age; these trends were specular in those from the most deprived quintile, with steady reductions for older ages. Conversely, proportions were rather constant with increasing age in Black individuals. Generally, among individuals from Black, South Asian, and other ethnic groups, the distribution across deprivation quintiles was similar between those with and without T2D across all age groups: proportion ratios for being from each of the deprivation quintiles (proportion with T2D divided by proportion without T2D) were close to 1 (Figure S5 & S6) – indicating no association between T2D status and deprivation. However, in individuals from Black and other ethnic groups from older age groups (generally beyond 60 years of age) there was a trend towards proportion ratios < 1 of being from the least deprived quintile, indicating a lower proportion of individuals from the least deprived quintile in individuals with type 2 diabetes compared to those without. This was most clear in individuals from other ethnic groups beyond age 70 years. There was no association between T2D status and the most deprived quintile of deprivation at these ages however. At other ages, and in South Asian individuals, proportion ratios were close to 1.

Conversely, in White individuals at most ages, the proportion from the least deprived quintile was marginally lower in individuals with T2D as compared to individuals without (proportion ratios < 1), with no consistent pattern in proportion ratios across age. Similarly, the proportion from the most deprived quintile was marginally higher in those with T2D (proportion ratios > 1) across most age groups. For example, In White women, proportion ratios of being from the least deprived quintile were lowest at age 18–24 years (0.97 [95% CI: 0.95–0.99]), and highest at age 40–44 years (1.03 [1.01–1.04]); for the most deprived quintile, these were 0.99 (0.97–1.01) at age 25–29 years and 1.08 (1.07–1.1) at age 45–49 years. In men, proportion ratios for the least deprived quintile ranged from 0.96 (0.93–0.99) at age 25–29 years to 1.00 (0.98–1.01) at 50–54 years; and for the most deprived quintile, ranged from 1.00 (0.97–1.02) at age 25–29 years to 1.07 (1.04–1.11) at age ≥ 85 years.

## Discussion

This study identified several novel findings.

Firstly, we identified that the proportion of individuals from minority ethnic groups is consistently higher in individuals with T2D, compared to individuals without T2D, in almost all age groups above 18 years. We originally hypothesised that ethnicity might be a stronger risk factor for T2D at a younger age, which would cause greater proportions from minority ethnic groups with younger age relative to controls. This was based on prior evidence showing that younger individuals with T2D in England are more likely to be from Black, South Asian and other ethnic groups than older individuals with T2D [16]. However, our data demonstrates that this pattern largely reflects the underlying demographics of the general population, rather than suggesting a particularly heightened risk of T2D at younger ages among these groups. Our findings could support policy makers and public health bodies in resource planning and preventative strategies, by suggesting that resources could be used to address the risk of T2D in individuals from minority ethnic groups regardless of age, as the association between ethnicity and EOT2D does not appear to warrant particularly heightened concern.

Secondly, our findings suggest that there could be a variable association between T2D status and deprivation across ethnicity. At most ages, in White individuals, those with T2D were more likely to be from the most deprived quintile of deprivation, and less likely to be from the least deprived quintile of deprivation than individuals without T2D, which also did not appear to be heightened at a younger age compared to an older age. Unexpectedly, in individuals from Black, South Asian or other ethnic groups, there were no clear associations between T2D status and deprivation at most ages, albeit a potential association between type 2 diabetes and lower levels of deprivation beyond age 60 years in individuals from Black and other ethnic groups. From an intersectional perspective, this could suggest that conventional measures of socioeconomic position may not capture the forms of disadvantage most relevant to T2D risk in minority ethnic groups. Structural inequalities and experiences of racism may influence health outcomes in ways that are not reflected by area-based deprivation indices, which may operate differently in comparison to White populations. Further work is needed to explore how structural and social processes contribute to these patterns.

Further reasons from the lack of association between T2D and deprivation in individuals from minority ethnic groups include the potential for misclassification bias from a greater prevalence of undiagnosed T2D in individuals from minority ethnic groups with high levels of socioeconomic deprivation. Indeed, a prior study identified that undiagnosed T2D is more prevalent in individuals from Black or South Asian ethnic groups [24]. Consistent with this, a delay in diagnosis of T2D in individuals from Black ethnic groups may also be suggested a higher HbA1c at diagnosis relative to other ethnic groups regardless of age, in large representative sample aged 16–50 years from CPRD [25]. In England, there are known barriers to accessing healthcare in individuals from Black, South Asian and other ethnic groups, which has been well described with cancer screening uptake for example [26, 27]; these barriers may be exacerbated by deprivation, such as through financial barriers to healthcare access including the need for unpaid time off, childcare, or transportation [24, 25]. In England, T2D screening is largely opportunistic [28], which may contribute to underdiagnosis among individuals from minority ethnic groups with high levels of socioeconomic deprivation that experience barriers in accessing healthcare. These systemic barriers to healthcare access could result in T2D not being captured in primary care EHRs, leading to a falsely low proportion of individuals from these groups in our T2D cohort. The potential misclassification bias in our study is supported by discrepancies between our findings and those from a UK Biobank study of 27,748 individuals with and 446,436 without T2D, which demonstrated a positive association between T2D status and deprivation across all ancestries, with stronger associations in individuals with African or Asian ancestry than European ancestry [19]. Further research is warranted to explore whether undiagnosed T2D may be more common in individuals from minority ethnic groups with high levels of deprivation, and whether this could mask associations between deprivation and T2D.

Despite this, there may be other factors that contribute to the lack of association between T2D and deprivation in individuals from minority ethnic groups, and the differences between our findings and the UK Biobank. Firstly, the UK Biobank may be affected by selection bias, as participants tend to have more favourable health profiles and lower levels of deprivation than the general population [29]. Additionally, lifestyle factors associated with deprivation may vary across ethnic groups in ways not captured by UK Biobank. Indeed, in the US, studies have consistently found either no association, or a decreased risk of obesity, with increasing levels of socioeconomic deprivation in Black individuals, particularly men, which differs from associations in White individuals [30–33]. The UKBB also used genetic ancestry, whereas the present study used ethnicity which could also contribute to differences. Additional explanations for the lack of association between T2D and deprivation include an increase in experienced discrimination in individuals that are more highly educated [18, 34], a proxy for lower deprivation, and a larger health impact of discrimination with lower deprivation [35]; and that deprivation may be protective of T2D status in minority ethnic groups in extreme forms, such as a subsistence diet; reflecting associations outside of the West [36]. Finally, the healthy migrant effect, whereby migrants may have lower T2D risk than the general population, could contribute to findings, especially if migrants are more likely to live in more deprived areas. The reasons for the varying association between T2D status and deprivation across ethnic groups clearly requires further investigation.

Our findings also partially reflect findings from the US. Using the ‘All of Us’ cohort, authors previously found that White and Asian individuals had a higher prevalence of T2D with increasing deprivation, whereas a negative association was observed among Black and Hispanic individuals [18]. Whilst we found similar findings with regards to the association in White individuals, the association between deprivation and T2D status in other ethnic groups appeared to differ across the studies as we found no association between T2D status and deprivation. Given that these studies occurred in different countries, there could be a multitude of reasons explaining their differences, including varying cultural factors, health behaviours, healthcare access, health systems, genetic ancestry, migration status, and socio-economic factors. Indeed, the discrepant associations between deprivation and T2D in minority ethnic groups may reflect the contrasting associations between ethnicity and mortality and complications between the US and UK [11].

### Strengths and limitations

The main strength of this study was the use of a very large multi-ethnic and nationally-representative cohort [37, 38] allowing us to explore granular variations ethnicity/deprivation across several characteristics including T2D status, age and sex. Indeed, exposures do not occur in isolation, and understanding intersections in disease risk is of the utmost importance in order to firstly understand an individual’s risk, and secondly to inform interventions to reduce health inequalities [39]. Using a stratification approach, rather than a model-based approach, allowed examination of associations across intersecting characteristics without constraining effects to prespecified parametric forms. Furthermore, whilst all real-world data are limited by potential inaccuracies in clinical coding, CPRD has demonstrated high correctness in T2D coding [40]. Having matching individuals with and without T2D for sex, age and primary care practice also allowed for robust comparisons of ethnicity and deprivation across groups.

Our study also has several limitations, namely our cohort extraction only included individuals with complete data for ethnicity and deprivation, excluding approximately 25% of individuals, which could affect external validity, particularly as there were proportionally more women and fewer people with T2D amongst individuals with missing data as compared to the primary cohort. Individuals from certain ethnic and deprivation groups may be more likely to be missing both demographics and type 2 diabetes status due to an unequal presence of barriers in accessing healthcare. Whilst this is a flaw of all real-world research investigating associations across deprivation and ethnicity, it could mean that data are not missing at random and could bias estimates. Furthermore, given this study relied on EHRs, our study does not reflect individuals with undiagnosed T2D, which could lead to ascertainment bias. As discussed, prior literature suggests this may particularly influence the proportion of individuals with T2D from Black ethnic groups [24, 25]. Our study was also cross-sectional, meaning that we were not able to examine temporal associations. We also used IMD to define deprivation, which is a local-area measure of deprivation and so may introduce ecological bias, furthermore, given the cross-sectional nature of this study, we also did not account for changes in IMD over time. Similarly, we pooled multiple ethnicity subgroups (e.g., Bangladeshi, Indian, Pakistani) to create crude larger groups (e.g., South Asian), which may mask differential granular patterns in ethnicity subgroups. The matching of primary care practice may have influenced the deprivation and ethnicity distribution in individuals without T2D if these factors were clustered. For example, individuals from the same primary care practice may be more likely to be from the same ethnic or deprivation group, compared to individuals from different practices. The effect would be to reduce the difference in the proportions across T2D status (proportion ratios closer the 1), this may be particularly concerning for deprivation findings, as in many groups, no association was observed. The potential for inadvertent partial matching of ethnicity/deprivation across T2D status also means that the magnitude of associations should be interpreted cautiously. Furthermore, the analysis did not consider calendar time. Results are likely to show the average proportions over the study period. Finally, like all observational studies, it is not possible to infer causality.

## Conclusion

In conclusion, our findings suggest that the higher proportion of individuals with T2D from Black, South Asian or other ethnic groups with a younger age largely reflect the demographic structure of the UK population without T2D. Although individuals from these backgrounds remain at increased risk of T2D, this does not appear to be particularly heightened at a younger age. We also demonstrate that White individuals with higher levels of deprivation are likely to be at increased risk of T2D, albeit only marginally, whereas these associations were absent in other ethnic groups. This raises important research questions as to whether T2D may be undiagnosed in individuals from minority ethnic groups with high levels of deprivation, which could be biasing our findings. Regardless, our data supports the need for strategies to prevent T2D in Black, South Asian or other ethnic groups, and in White individuals with higher levels of deprivation, regardless of age. Finally, our results highlight the relevance of accounting for the dynamic changes in the sociodemographic structure of the general population when investigating the complex intersectionality between ethnicity and deprivation in the risk of T2D.

## Electronic supplementary material

Below is the link to the electronic supplementary material.

Supplementary Material 1

## Data Availability

The patient-level data used in this study cannot be shared but can be accessed via CPRD. All codes used in the analysis and all estimates from the results are publicly available on GitHub (jgoldney).

## Declarations

### Twitter summary

High proportions of young people with T2D from Black, South Asian or other ethnic groups largely reflects patterns in general population. Associations between T2D and deprivation vary across ethnic groups.
