# Supplementary material for: Associations of age at type 2 diabetes diagnosis with ethnicity and socioeconomic deprivation: observational study in 11.5 million people in England
Source: Int J Equity Health. 2026 Jun 10;25:200. doi: 10.1186/s12939-026-02924-w (PMC13501700; doi:10.1186/s12939-026-02924-w)
Supplement: Supplementary file 1 [file 12939_2026_2924_MOESM1_ESM.docx]

**Associations of age at type 2 diabetes diagnosis with ethnicity and socioeconomic deprivation: observational study in 11.5 million people in England**

**Supplementary material**

Contents

[**Supplementary Table S1:** Number of individuals by diabetes status across subgroups of ethnicity	2](#_Toc221895439)

[**Supplementary Table S2:** Characteristics of the cohort with missing ethnicity	3](#_Toc221895440)

[**Supplementary Figure S1:** Cohort definition	4](#_Toc221895441)

[**Supplementary Figure S2:** The proportion of women and men from each ethnic group, across diabetes status, age and the second, third and fourth quintiles of deprivation	5](#_Toc221895442)

[**Supplementary Figure S3:** Proportion ratios of being from each ethnic group comparing people with vs without type 2 diabetes, by sex, age and deprivation quintile	6](#_Toc221895443)

[**Supplementary Figure S4:** The proportion of women and men from the second, third and fourth quintile of deprivation, by diabetes status, age and ethnicity	7](#_Toc221895444)

[**Supplementary Figure S5:** Proportion ratios of being from the least or most deprived quintile comparing people with vs without type 2 diabetes, by sex, age and ethnicity	8](#_Toc221895445)

[**Supplementary Figure S6:** Proportion ratios of being from the middle three quintiles of deprivation comparing people with vs without type 2 diabetes, by sex, age and ethnicity	9](#_Toc221895446)

[**RECORD checklist**	10](#_Toc221895447)

# **Supplementary Table S1:** Number of individuals by diabetes status across subgroups of ethnicity

|  | | **Individuals with type 2 diabetes** | | **Individuals without type 2 diabetes** | |
| --- | --- | --- | --- | --- | --- |
|  |  | **Women** | **Men** | **Women** | **Men** |
| **Black ethnicity** | **Black African** | 44,896 (3.12) | 27,702 (2.24) | 132,146 (2.68) | 69,353 (1.71) |
|  | **Black Caribbean** | 34,727 (2.41) | 23,439 (1.90) | 82,937 (1.68) | 55,668 (1.37) |
|  | **Black Other** | 20,184 (1.40) | 14,336 (1.16) | 54,494 (1.10) | 38,877 (0.96) |
| **South Asian ethnicity** | **Bangladeshi** | 19,026 (1.32) | 12,912 (1.04) | 38,028 (0.77) | 24,255 (0.60) |
|  | **Indian** | 46,354 (3.22) | 37,277 (3.01) | 109,714 (2.22) | 75,239 (1.86) |
|  | **Pakistani** | 32,265 (2.24) | 24,483 (1.98) | 68,105 (1.38) | 48,272 (1.19) |
| **White ethnicity** | **White** | 1,153,278 (80.12) | 1,024,329 (82.84) | 4,174,589 (84.64) | 3,551,528 (87.58) |
| **Other ethnicities** | **Chinese** | 7,332 (0.51) | 4,637 (0.38) | 26,907 (0.55) | 13,383 (0.33) |
|  | **Other Asian** | 26,575 (1.85) | 24,661 (1.99) | 67,142 (1.36) | 54,003 (1.33) |
|  | **Mixed** | 16,119 (1.12) | 10,472 (0.85) | 55,866 (1.13) | 32,659 (0.81) |
|  | **Other** | 38,754 (2.69) | 32,281 (2.61) | 122,312 (2.48) | 91,870 (2.27) |

Data are presented as number (% of column-wise total).

**Supplementary Table S2:** Characteristics of the cohort with missing ethnicity

|  | | **Women** | | **Men** | |
| --- | --- | --- | --- | --- | --- |
|  |  | **Individuals with type 2 diabetes** | **Individuals without type 2 diabetes** | **Individuals with type 2 diabetes** | **Individuals without type 2 diabetes** |
| **No. of people** | | 318,903 (6.7) | 1,762,291 (36.8) | 405,183 (8.5) | 2,307,788 (48.1) |
| **Median age (years)** | | 49 [33, 62] | 45 [31, 59] | 47 [38, 58] | 46 [36, 57] |
| **Age categories (years)** | **18-25** | 31,247 (9.8%) | 207,308 (11.8%) | 17,461 (4.3%) | 119,722 (5.2%) |
|  | **25-29** | 31,266 (9.8%) | 191,550 (10.9%) | 25,148 (6.2%) | 158,399 (6.9%) |
|  | **30-34** | 25,767 (8.1%) | 167,020 (9.5%) | 34,655 (8.6%) | 216,013 (9.4%) |
|  | **35-39** | 20,697 (6.5%) | 142,378 (8.1%) | 40,239 (9.9%) | 249,378 (10.8%) |
|  | **40-44** | 25,525 (8.0%) | 160,875 (9.1%) | 54,633 (13.5%) | 317,419 (13.8%) |
|  | **45-49** | 28,338 (8.9%) | 158,137 (9.0%) | 52,897 (13.1%) | 298,228 (12.9%) |
|  | **50-54** | 31,352 (9.8%) | 160,301 (9.1%) | 49,620 (12.2%) | 267,568 (11.6%) |
|  | **55-59** | 30,974 (9.7%) | 145,061 (8.2%) | 41,112 (10.1%) | 211,425 (9.2%) |
|  | **60-64** | 27,762 (8.7%) | 121,673 (6.9%) | 32,698 (8.1%) | 161,770 (7.0%) |
|  | **65-69** | 22,638 (7.1%) | 96,035 (5.4%) | 24,561 (6.1%) | 117,755 (5.1%) |
|  | **70-74** | 16,977 (5.3%) | 73,635 (4.2%) | 15,518 (3.8%) | 79,313 (3.4%) |
|  | **75-79** | 11,281 (3.5%) | 52,591 (3.0%) | 8,565 (2.1%) | 50,253 (2.2%) |
|  | **80-84** | 7,566 (2.4%) | 38,574 (2.2%) | 4,810 (1.2%) | 31,911 (1.4%) |
|  | **≥85** | 7,513 (2.4%) | 47,153 (2.7%) | 3,266 (0.8%) | 28,634 (1.2%) |
| **Quintile of deprivation** | **1^st^ - Least deprived** | 56,131 (17.6%) | 311,258 (17.7%) | 72,477 (17.9%) | 402,916 (17.5%) |
|  | **2^nd^** | 56,671 (17.8%) | 323,911 (18.4%) | 70,194 (17.3%) | 413,405 (17.9%) |
|  | **3^rd^** | 64,255 (20.1%) | 359,134 (20.4%) | 79,882 (19.7%) | 461,423 (20.0%) |
|  | **4^th^** | 81,156 (25.4%) | 447,683 (25.4%) | 101,868 (25.1%) | 578,523 (25.1%) |
|  | **5^th^ - Most deprived** | 60,199 (18.9%) | 312,473 (17.7%) | 80,203 (19.8%) | 441,462 (19.1%) |
|  | **Missing** | 491 (0.2%) | 7,832 (0.4%) | 559 (0.1%) | 10,059 (0.4%) |

Data are presented as number (%) for categorical variables column-wise, excluding no. of people which is presented row-wise, and as median (interquartile range) for continuous variables.

Age indicates the age at diagnosis in individuals with type 2 diabetes and the matched age in those without type 2 diabetes.

# **Supplementary Figure S1:** Cohort definition

**HES:** Hospital Episode Statistics; **ONS:** Office for National Statistics; **CPRD:** Clinical Practice Research Datalink; **IMD:** 2019 Index of Multiple Deprivation.

Some observations could have missing data for both IMD and ethnicity.

Both CPRD GOLD and Aurum were used as they reflect primary care practices using different electronic health record systems, and including both augments the total sample size. The different sampling period up to 29/3/2021 reflects the latest possible date available at the time of data extraction.

# **Supplementary Figure S2:** The proportion of women and men from each ethnic group, across diabetes status, age and the second, third and fourth quintiles of deprivation

Each plot shows the proportion of women (left panels) and men (right panels) in each ethnicity group across type 2 diabetes (T2D) status (individuals with vs without T2D: circles vs triangles, respectively), quintiles of deprivation (quntile 2, least deprived, to quintile 4, most deprived) and age at T2D diagnosis (x-axis).

Note the Y axis range varies by outcome. Estimates are shown with 95% confidence intervals (bars). Dotted lines between estimates were added for visual aid and age was used as a categorical variable, not a continuous.

# **Supplementary Figure S3:** Proportion ratios of being from each ethnic group comparing people with vs without type 2 diabetes, by sex, age and deprivation quintile

Each plot shows the proportion ratio for each specific ethnic group in women (left panels) or men (right panels) comparing individuals with vs without type 2 diabetes by age at diagnosis (x-axis) and deprivation.

Proportion ratios are calculated by dividing the proportion from a Black ethnic group with type 2 diabetes divided by the proportion from a Black ethnic group without type 2 diabetes, for example.

The Y axis is plotted on the logarithmic scale and the range varies by outcome. Estimates are shown with 95% confidence intervals (bars). Dotted lines between estimates were added for visual aid and age was used as a categorical variable, not a continuous.

# **Supplementary Figure S4:** The proportion of women and men from the second, third and fourth quintile of deprivation, by diabetes status, age and ethnicity

Each plot shows the proportion of women (left panels) or men (right panels) from the second (less deprived), third and fourth (more deprived) quintile of deprivation group across type 2 diabetes (T2D) status (individuals with vs without T2D: circles vs triangles, respectively) and age at T2D diagnosis (x-axis).

Note the Y axis range varies by outcome. Estimates are shown with 95% confidence intervals (bars). Dotted lines between estimates were added for visual aid and age was used as a categorical variable, not a continuous.

# **Supplementary Figure S5:** Proportion ratios of being from the least or most deprived quintile comparing people with vs without type 2 diabetes, by sex, age and ethnicity

Each plot shows the proportion ratio of being from deprivation quintile 1 (least deprived, red) and quintile 5 (most deprived, green) across sex and ethnicity.

Proportion ratios are calculated by dividing the proportion from deprivation quintile 1 with type 2 diabetes divided by the proportion from deprivation quintile 1 without type 2 diabetes, for example.

The Y axis is plotted on the logarithmic scale and the range varies by outcome. Estimates are shown with 95% confidence intervals (bars). Dotted lines between estimates were added for visual aid and age was used as a categorical variable, not a continuous.

# **Supplementary Figure S6:** Proportion ratios of being from the middle three quintiles of deprivation comparing people with vs without type 2 diabetes, by sex, age and ethnicity

Each plot shows the proportion ratio of being from deprivation quintiles 2-4 across sex and ethnicity. A lower quintile represents a lower level of deprivation.

Proportion ratios are calculated by dividing the proportion from deprivation quintile 2 with type 2 diabetes divided by the proportion from deprivation quintile 2 without type 2 diabetes, for example.

The Y axis is plotted on the logarithmic scale and the range varies by outcome. Estimates are shown with 95% confidence intervals (bars). Dotted lines between estimates were added for visual aid and age was used as a categorical variable, not a continuous.

# **RECORD checklist**

A checklist of items, extended from the STROBE statement, that should be reported in observational studies using routinely collected health data.

|  | **Item No.** | **STROBE items** | **Location in manuscript where items are reported** | **RECORD items** | **Location in manuscript where items are reported** |
| --- | --- | --- | --- | --- | --- |
| **Title and abstract** | | | | | |
|  | 1 | (a) Indicate the study’s design with a commonly used term in the title or the abstract (b) Provide in the abstract an informative and balanced summary of what was done and what was found | Title & Abstract, page 1, 2 | RECORD 1.1: The type of data used should be specified in the title or abstract. When possible, the name of the databases used should be included.  RECORD 1.2: If applicable, the geographic region and timeframe within which the study took place should be reported in the title or abstract.  RECORD 1.3: If linkage between databases was conducted for the study, this should be clearly stated in the title or abstract. | Title & Abstract, page 1, 2 |
| **Introduction** | | | | | |
| Background rationale | 2 | Explain the scientific background and rationale for the investigation being reported | Background, page 5-6 |  |  |
| Objectives | 3 | State specific objectives, including any prespecified hypotheses | Background, page 6 |  |  |
| **Methods** | | | | | |
| Study Design | 4 | Present key elements of study design early in the paper | Research design and methods, page 7 |  |  |
| Setting | 5 | Describe the setting, locations, and relevant dates, including periods of recruitment, exposure, follow-up, and data collection | Research design and methods, page 7 |  |  |
| Participants | 6 | *Cross-sectional study* - Give the eligibility criteria, and the sources and methods of selection of participants | Research design and methods, page 7-8, **Supplementary Figure S1** | RECORD 6.1: The methods of study population selection (such as codes or algorithms used to identify subjects) should be listed in detail. If this is not possible, an explanation should be provided.  RECORD 6.2: Any validation studies of the codes or algorithms used to select the population should be referenced. If validation was conducted for this study and not published elsewhere, detailed methods and results should be provided.  RECORD 6.3: If the study involved linkage of databases, consider use of a flow diagram or other graphical display to demonstrate the data linkage process, including the number of individuals with linked data at each stage. | Research design and methods, page 7-8, **Supplementary Figure S1** |
| Variables | 7 | Clearly define all outcomes, exposures, predictors, potential confounders, and effect modifiers. Give diagnostic criteria, if applicable. | Research design and methods, page 8 | RECORD 7.1: A complete list of codes and algorithms used to classify exposures, outcomes, confounders, and effect modifiers should be provided. If these cannot be reported, an explanation should be provided. | Research design and methods, page 8 |
| Data sources/ measurement | 8 | For each variable of interest, give sources of data and details of methods of assessment (measurement).  Describe comparability of assessment methods if there is more than one group | Research design and methods, page 8 |  |  |
| Bias | 9 | Describe any efforts to address potential sources of bias | Research design and methods, page 8-9 |  |  |
| Study size | 10 | Explain how the study size was arrived at | **Supplementary Figure S1** |  |  |
| Quantitative variables | 11 | Explain how quantitative variables were handled in the analyses. If applicable, describe which groupings were chosen, and why | Statistical analysis, page 8-9 |  |  |
| Statistical methods | 12 | (a) Describe all statistical methods, including those used to control for confounding  (b) Describe any methods used to examine subgroups and interactions  (c) Explain how missing data were addressed  *Cross-sectional study* - If applicable, describe analytical methods taking account of sampling strategy  (e) Describe any sensitivity analyses | Statistical analysis, page 8-9 |  |  |
| Data access and cleaning methods |  | .. |  | RECORD 12.1: Authors should describe the extent to which the investigators had access to the database population used to create the study population.  RECORD 12.2: Authors should provide information on the data cleaning methods used in the study. | - |
| Linkage |  | .. |  | RECORD 12.3: State whether the study included person-level, institutional-level, or other data linkage across two or more databases. The methods of linkage and methods of linkage quality evaluation should be provided. | Research design and methods, page 8 |
| **Results** | | | | | |
| Participants | 13 | (a) Report the numbers of individuals at each stage of the study (*e.g.*, numbers potentially eligible, examined for eligibility, confirmed eligible, included in the study, completing follow-up, and analysed)  (b) Give reasons for non-participation at each stage.  (c) Consider use of a flow diagram | Cohort characteristics, page 10, Supplementary Figure S1 | RECORD 13.1: Describe in detail the selection of the persons included in the study (*i.e.,* study population selection) including filtering based on data quality, data availability and linkage. The selection of included persons can be described in the text and/or by means of the study flow diagram. | Cohort characteristics, page 10, Supplementary Figure S1 |
| Descriptive data | 14 | (a) Give characteristics of study participants (*e.g.*, demographic, clinical, social) and information on exposures and potential confounders  (b) Indicate the number of participants with missing data for each variable of interest | Cohort characteristics, page 10, Supplementary Figure S1 |  |  |
| Outcome data | 15 | *Cross-sectional study* - Report numbers of outcome events or summary measures | Cohort characteristics, page 10 |  |  |
| Main results | 16 | (a) Give unadjusted estimates and, if applicable, confounder-adjusted estimates and their precision (e.g., 95% confidence interval). Make clear which confounders were adjusted for and why they were included  (b) Report category boundaries when continuous variables were categorized  (c) If relevant, consider translating estimates of relative risk into absolute risk for a meaningful time period | Results, page 10-16 |  |  |
| Other analyses | 17 | Report other analyses done—e.g., analyses of subgroups and interactions, and sensitivity analyses | Results, page 10-16 |  |  |
| **Discussion** | | | | | |
| Key results | 18 | Summarise key results with reference to study objectives | Discussion, page 17 |  |  |
| Limitations | 19 | Discuss limitations of the study, taking into account sources of potential bias or imprecision. Discuss both direction and magnitude of any potential bias | Discussion, page 20 | RECORD 19.1: Discuss the implications of using data that were not created or collected to answer the specific research question(s). Include discussion of misclassification bias, unmeasured confounding, missing data, and changing eligibility over time, as they pertain to the study being reported. | Discussion, page 20 |
| Interpretation | 20 | Give a cautious overall interpretation of results considering objectives, limitations, multiplicity of analyses, results from similar studies, and other relevant evidence | Discussion, page 17-21 |  |  |
| Generalisability | 21 | Discuss the generalisability (external validity) of the study results | Discussion, page 17-19 |  |  |
| **Other Information** | | | | | |
| Funding | 22 | Give the source of funding and the role of the funders for the present study and, if applicable, for the original study on which the present article is based | Acknowledgements, page 22 |  |  |
| Accessibility of protocol, raw data, and programming code |  | .. |  | RECORD 22.1: Authors should provide information on how to access any supplemental information such as the study protocol, raw data, or programming code. | Acknowledgements, page 23 |

*Reference: Benchimol EI, Smeeth L, Guttmann A, Harron K, Moher D, Petersen I, Sørensen HT, von Elm E, Langan SM, the RECORD Working Committee. The REporting of studies Conducted using Observational Routinely-collected health Data (RECORD) Statement. *PLoS Medicine.* 2015 Oct 6;12(10):e1001885.

Page numbers refer to the original, word document submission.
